# The Queen at St. Bartholomew's

**Published:** 1921-02-26

**Authors:** 


### The Queen at St. Bartholomew's.

A milestone in the history of St. Bartholomew's Hospital was passed on February 17 when H.M. Queen Mary laid the foundation-stone of the nurses' home which is to bear her name. Some fifteen hundred persons witnessed the ceremony, which was graced by the presence of Majesty, the Royal President and Princess Mary. A monster marquee on part of the site of the new Home had been erected, and here on a raised platform, after the reception of Queen Mary by the Prince of Wales and certain presentations, the ceremony of the day took place. The Bishop of London said the prayers and His Royal Highness read an address to the Queen, who then performed the ceremony and declared the stone "well and truly laid." A casket containing the St. Bartholomew's Hospital last report and the *Journal* of the Nurses' League was buried beneath the stone.

The Prince, in his subsequent speech, spoke with pleasure of the great financial support which the hospital has recently received, including individual donations of £25,000 and £10,000, besides other large sums from public bodies and money collected by various people. We hope this munificence may continue, so that when the first block of the new Home is erected the funds to proceed with the remaining blocks will be in hand. The need for the whole is self-evident, and to those who may be ignorant of the present accommodation provided for St. Bartholomew's nurses we would urge a visit to the hospital, where in old, mean, and small houses, crowded together on an unsuitable site, without conveniences or adequate hygienic arrangements, the nursing staff of one of our greatest Metropolitan hospitals is housed. Seeing is believing, and such a visit should prove of inestimable value to the visitor and to the finances of the hospital. The Appeal Committee should photograph these mean quarters and let them make their own appeal. In our last issue, page 483, we published an account of the new accommodation which has been planned. Its aim is simplicity and practicability, with healthy conditions of housing, comfort, convenience, and economy of administration. This is the minimum to which any nursing staff is entitled.

To use the President's own words, Her Majesty's presence was a further testimony of the solicitude she has always shown for the health and comfort of those concerned in the nursing of the sick. The deep sense of gratitude inspired by Her Majesty's gracious and sympathetic interest in nursing matters is due not merely to the glamour which surrounds the throne, but to the deep knowledge and clear-sighted observation which the Queen brings to bear on whatever affects the well-being of nurses. The long-continued trials to which St. Bartholomew's nurses have been exposed cannot fail to be known to Her Majesty, and, therefore, it was, we may well believe, a particular gratification to her to inaugurate the new era. That the Queen's presence on this occasion will awaken and stimulate an interest which ought to be felt by everyone in this country in speedily bringing this good work to a conclusion is our earnest hope.

